# Utilizing a Disposable Sensor with Polyaniline-Doped Multi-Walled Carbon Nanotubes to Enable Dopamine Detection in Ex Vivo Mouse Brain Tissue Homogenates

**DOI:** 10.3390/bios14060262

**Published:** 2024-05-21

**Authors:** Thenmozhi Rajarathinam, Sivaguru Jayaraman, Jaeheon Seol, Jaewon Lee, Seung-Cheol Chang

**Affiliations:** 1 Engineering Research Center for Color-Modulated Extra-Sensory Perception Technology, Pusan National University, Busan 46241, Republic of Korea; thenmozhi@pusan.ac.kr; 2 Department of Cogno-Mechatronics Engineering, College of Nanoscience and Nanotechnology, Pusan National University, Busan 46241, Republic of Korea; sivaguru@pusan.ac.kr; 3 BIT Convergence-Based Innovative Drug Development Targeting Metainflammation, Department of Pharmacy, College of Pharmacy, Pusan National University, Busan 46241, Republic of Korea; tjfwogjs@pusan.ac.kr (J.S.); neuron@pusan.ac.kr (J.L.); 4 Department of Pharmacy, College of Pharmacy, Pusan National University, Busan 46241, Republic of Korea

**Keywords:** disposable sensor, dopamine, polyaniline, multi-walled carbon nanotubes, ex vivo detection

## Abstract

Disposable sensors are inexpensive, user-friendly sensing tools designed for rapid single-point measurements of a target. Disposable sensors have become more and more essential as diagnostic tools due to the growing demand for quick, easy-to-access, and reliable information related to the target. Dopamine (DA), a prevalent catecholamine neurotransmitter in the human brain, is associated with central nervous system activities and directly promotes neuronal communication. For the sensitive and selective estimation of DA, an enzyme-free amperometric sensor based on polyaniline-doped multi-walled carbon nanotubes (PANI-MWCNTs) drop-coated disposable screen-printed carbon electrodes (SPCEs) was fabricated. This PANI-MWCNTs-2/SPCE sensor boasts exceptional accuracy and sensitivity when working directly with ex vivo mouse brain homogenates. The sensor exhibited a detection limit of 0.05 μM (S/N = 3), and a wide linear range from 1.0 to 200 μM. The sensor’s high selectivity to DA amidst other endogenous interferents was recognized. Since the constructed sensor is enzyme-free yet biocompatible, it exhibited high stability in DA detection using ex vivo mouse brain homogenates extracted from both Parkinson’s disease and control mice models. This research thus presents new insights into understanding DA release dynamics at the tissue level in both of these models.

## 1. Introduction

In the human brain, dopamine (DA), a vasomodulatory neurotransmitter, plays a major role in regulating many aspects of the brain’s circuitry by sending impulses and controlling the brain’s physiological processes [1]. The physiological levels of DA in healthy human serum lie between 0.01 and 10 μM. Several neurological diseases, including Alzheimer’s disease and Parkinson’s disease (PD), schizophrenia, Tourette’s syndrome, attention deficit hyperactivity disorder, and pituitary tumors, may be exacerbated by DA dysregulation or changes [2,3]. Among these, PD is an incurable neurodegenerative disease, which is characterized by the progressive loss of substantia nigra dopaminergic neurons in the affected brain areas. Reduced DA content in the substantia nigra and striatum triggers the onset of PD. Thus, it is paramount to understand the influence of dopaminergic neurotransmission on whole-brain hemodynamics. Notably, Mark Wightman’s and Jill Venton’s research groups have extensively explored DA dynamics in several models including single-cell, ex vivo, and in vivo models using carbon nanotubes customized carbon-fiber microelectrodes [4,5,6,7,8,9]. Since measuring DA still presents significant challenges, developing a sensitive platform for DA detection is important to investigate the direct connection between DA dynamics and PD [10].

Until today, the enzyme-linked immunoassay [11], fluorimetry [12], UV/Visible spectrophotometry [13], and ionic chromatography [14] methods are frequently used to determine DA levels. These methods are expensive, time-consuming, and laborious, making them unsuitable for the rapid detection of DA. In addition, electrochemical sensors have been applied extensively in the detection of DA due to their exceptional characteristics, such as their high sensitivity in biological samples, quick response times, and ease of use [15]. Disposable electrochemical sensors also enable contamination and recalibration-free methods [16] for rapid and trace detection of DA [17,18].

The electrochemical response towards DA is highly dependent on the nanomaterial’s composition and surface characteristics. Multi-walled carbon nanotubes (MWCNTs) possess a large specific surface area, high electronic conductivity, little surface fouling, and chemical stability, which all have a special electrocatalytic impact on DA detection [19,20]. The implicit electrocatalytic activity of MWCNTs enables the discrimination of DA from AA and UA [21]. Conductive polymers modify the charge distribution of MWCNTs in the band structure and tune the Fermi energy level, thus improving the electrocatalytic activity of MWCNTs [22]. The combination of PANI polymers and MWCNTs allows tunable conductivity through protonation or charge-transfer doping and increased surface-active sites, which further enhance the sensing performance of the nanocomposite compared to that of its individual constituents.

**Scheme 1:**
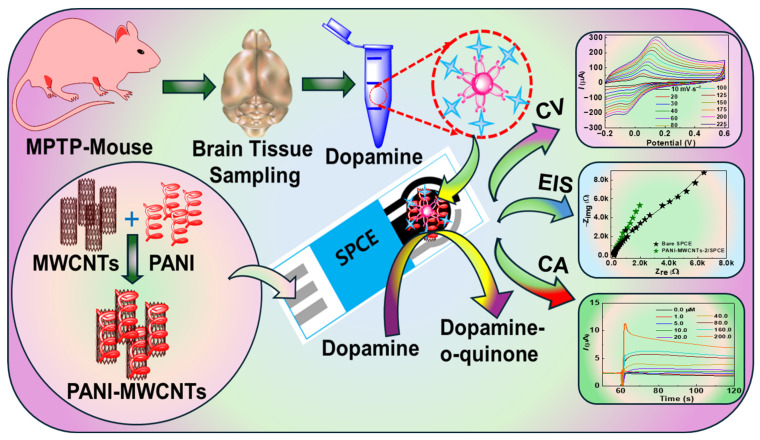
Simple representation of the synthesized PANI-MWCNT nanocomposites, the fabricated PANI-MWCNTs/SPCE sensor, and its application in ex vivo mouse brain tissues for rapid DA detection.

In this work, we report a rapid, disposable, cost-effective, and enzyme-free electrochemical sensor based on MWCNTs with a PANI nanocomposite, abbreviated as PANI-MWCNTs-2, for the detection of DA. A charge-transfer reaction between the PANI and MWCNTs is facilitated due to the overlapping of π-electrons between the graphitic MWCNT structure and aromatic ring of the PANI. The synthesized PANI-MWCNTs-2 provides a large specific surface area, which assures sufficient electrochemical properties of the nanocomposite. The PANI-MWCNTs-2 endows the disposable screen-printed carbon electrode (SPCE) with outstanding mechanical stability and electrocatalytic activity favorable towards DA detection in biological targets. The PANI-MWCNTs-2 sensor exhibits a wide linear range from 1.0 to 200 μM, a sensitivity of 2.3 µA mM^−1^ cm^−2^, and a detection limit of 0.05 μM. Also, the nitrogen and sulfide functionalities in the PANI polymer further enhance the PANI-MWCNTs-2/SPCE sensor’s properties towards selective DA detection, without the need for specific enzyme catalysts. A simple graphical representation of the designed sensor and its application in the ex vivo mouse brain tissue homogenates is illustrated in Scheme 1.

## 2. Materials and Methods

### 2.1. Materials

The following chemicals were received from Sigma–Aldrich (Seoul, Republic of Korea): ammonia aqueous solution (25–28%), aniline (99%), ammonium peroxydisulphate (APS) (Extrapure, 99%), hydrochloric acid (HCl, 37%), concentrated sulfuric acid (H_2_SO_4_, 98%), ethanol (99.5% purity), chloroform, MWCNTs (>95 wt%), potassium ferri and ferrocyanides [Fe(CN)_6_]^3−/4−^, potassium chloride (KCl), dopamine hydrochloride (DA), glucose, glutamate (Glu), norepinephrine (NE), serotonin (5–HT), ascorbic acid (AA), and uric acid (UA). Phosphate buffers (0.1 M) were prepared following a standard protocol reported elsewhere [23], and all solutions were prepared utilizing 18.2 MΩ cm water. Only aniline was distilled before use; all other chemicals were utilized exactly as they were supplied (analytical grade). The MWCNTs were refluxed for 3 h in 1 M HCl, washed using 18.2 MΩ cm water, and dried in a vacuum oven. SPCEs (Model No. C11L) were purchased from Metrohm DropSens (Oveido, Spain).

### 2.2. Instrumentation and Measurements

Details on the instruments utilized and the measurement conditions are given in Section S1 of the Supplementary Materials.

### 2.3. Synthesis of the PANI-MWCNTs

#### 2.3.1. Synthesis of PANI

PANI was synthesized following the self-stabilized dispersion polymerization (SSDP) method, as reported earlier [24]. For this SSDP method, distilled aniline (0.50 mL) was dissolved in 1.0 M HCl (30.0 mL) and polymerization was initiated with the addition of APS (0.60 g) in the presence of an organic solvent, i.e., chloroform. The reaction mixture was kept in an ice bath (0–5 °C) while being stirred for 12 h. The PANI product was then filtered; washed with ethanol, followed by 18.2 MΩ cm water; and vacuum-dried at 60 °C overnight.

#### 2.3.2. Synthesis of PANI-MWCNTs Using an In Situ Polymerization Method

**Scheme 2:**
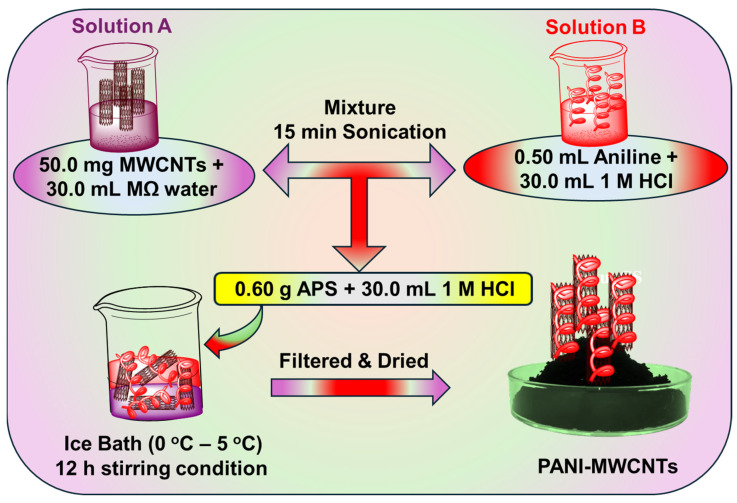
Schematic of the in situ SSDP method for the synthesis of PANI-MWCNT nanocomposites.

The utilized MWCNTs had an outer diameter of 20–30 nm, while their inner diameter was 5–10 nm and their lengths were 10–30 μm. The PANI-MWCNT nanocomposites were prepared using two different loading percentages (2.5 wt% and 5.0 wt%) using the same SSDP method. Briefly, to prepare a composite with a 5.0 wt% loading of MWCNTs, 50.0 mg MWCNTs were dispersed in 18.2 MΩ cm water (30.0 mL) via sonication, and this was labeled as Solution A. A 0.50 mL amount of aniline was dissolved in 1 M HCl (30.0 mL) and sonicated for 15 min, and this was labeled as Solution B. Solution A and B were mixed, and ammonium persulfate (0.60 g) dissolved in 1 M HCl (30.0 mL) was then added dropwise and maintained for 12 h under constant stirring in an ice bath (0 and 5 °C) for complete in situ polymerization reactions. The precipitate was washed with ethanol and 18.2 MΩ cm water until the pH value reached 7.0. Then, the greenish-black powder products were vacuum-dried at 60 °C overnight. The same procedure was followed to prepare 2.5 wt% PANI-MWCNTs. A schematic of the synthesis procedure for the 5.0 wt% PANI-MWCNTs is displayed in Scheme 2.

### 2.4. Fabrication of the PANI-MWCNTs-2/SPCE Sensor

The SPCEs were drop-coated with 6.0 µL of the synthesized PANI-MWCNTs and dried at room temperature (25 °C). The presence of the PANI polymer in the PANI-MWCNTs-2 nanocomposite sensor enabled the formation of a stable and highly sensitive layer for DA detection.

### 2.5. Preparation of Ex Vivo Brain Tissue Homogenates from PD and Control Models

The functionality of the sensor was evaluated by analyzing ex vivo brain tissue homogenates from mouse models of PD induced by 1-methyl-4-phenyl-1,2,3,6-tetrahydropyridine (MPTP), alongside control models. MPTP is a well-established neurotoxin that induces PD by causing damage to dopaminergic neurons in the nigrostriatal pathway, resulting in a significant decrease in DA levels. We obtained six-week-old C57BL/6 N mice weighing 20–25 g from Daehan Bio-Link (Seoul, Republic of Korea). The mice were randomly divided into two groups: MPTP and control (PBS) groups, and they were maintained under controlled conditions with a temperature range of 20–23 °C and a 12 h light–dark cycle and provided with food and water ad libitum. After a 4-week acclimatization, MPTP was intraperitoneally (*i.p.*) administered to mice in the MPTP group at a dosage of 30 mg/kg once daily for 5 consecutive days, while the control group received an equivalent volume of 0.1 M phosphate-buffered saline (PBS). Euthanasia was performed 72 h after MPTP/PBS administration.

The animal protocol was reviewed and approved by the Pusan National University Institutional Animal Care Committee (approval number: PNU-2021-2961) to ensure that ethical standards were upheld throughout the experiment.

## 3. Results and Discussion

### 3.1. Physicochemical Characterizations

**Figure 1:**
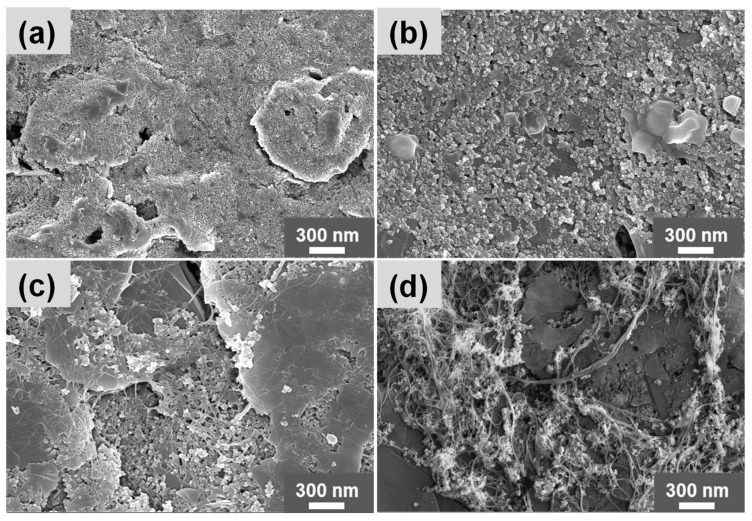
FE–SEM images of the modified sensors: (**a**) bare SPCE, (**b**) PANI/SPCE, (**c**) PANI-MWCNTs-1/SPCE, and (**d**) PANI-MWCNTs-2/SPCE. Scale bar: 300 nm.

**Figure 2:**
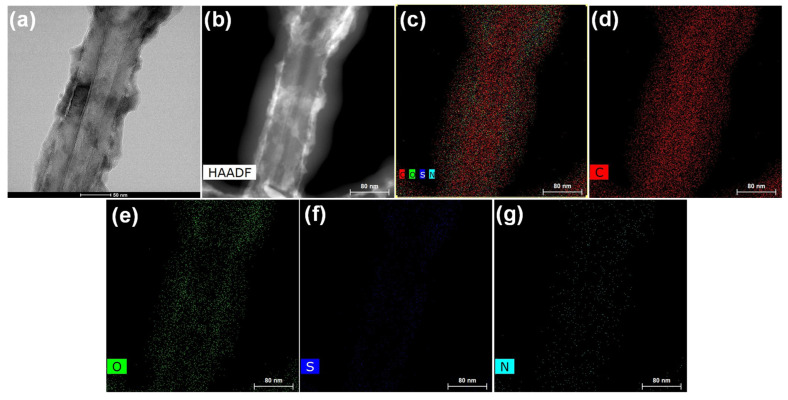
(**a**) FETEM image of PANI-MWCNTs-1 on a 50 nm scale bar, (**b**) HAADF scan image, (**c**) merged HAADF scan image illustrating the presence of C, O, S, and N elements, (**d**–**g**) each individual element on a 80 nm scale bar.

**Figure 3:**
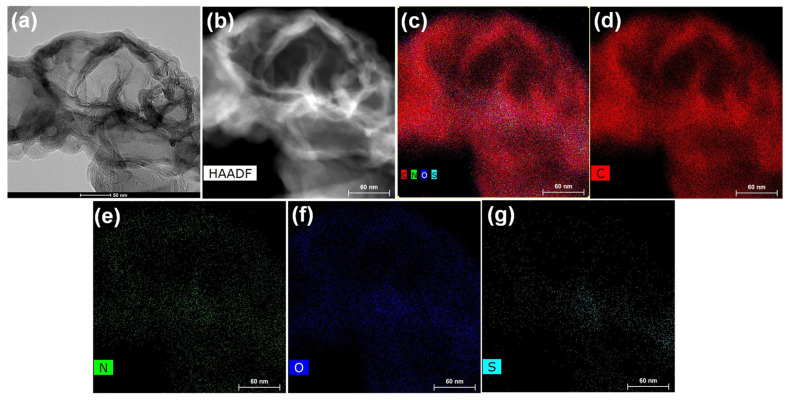
(**a**) FETEM image of PANI-MWCNTs-2 on a 50 nm scale bar, (**b**) HAADF scan image, (**c**) merged HAADF scan image showing C, N, O, and S elements, (**d**–**g**) each individual element on a 60 nm scale bar.

The physicochemical properties of the bare SPCE, PANI/SPCE, PANI-MWCNTs-1/SPCE, and PANI-MWCNTs-2/SPCE were explored using field emission scanning electron microscopy (FE–SEM) as shown in Figure 1, Figure 2 and Figure 3. The bare SPCE (Figure 1a) displays a typical graphite-like morphology with rough surfaces, while the PANI/SPCE (Figure 1b) exhibits a granular morphology with a much smoother surface. Figure 1c,d reveal the presence of both a granular and tubular morphology, due to the combination of the PANI and MWCNTs. The tubular morphology evinces a typical morphology characteristic of MWCNTs. The intermingled ropes/tubes with smooth surfaces show the MWCNTs’ lengths up to a few microns.

The transmission electron microscopy (TEM) image in Figure 2a shows layers of a uniform wrapping/coating of PANI on the tubular surface of the MWCNTs. The high-angle annular dark-field scanning transmission electron microscopy (HAADF-STEM) image (Figure 2b) and the merged energy-dispersive X-ray spectrometry (EDX) image (Figure 2c) of PANI-MWCNTs-1 illustrate the presence of individual elements C, O, S, and N (Figure 2d–g).

Similarly, PANI-MWCNTs-2 (Figure 3a–g) shows the presence of C, O, S, and N elements with slight variations in their atomic percentages. The atomic% values of the C, O, S, and N elements in the PANI-MWCNTs-1 account for 92.8, 2.7, 1.8, and 2.7%, while the atomic% values of C, O, S, and N in PANI-MWCNTs-2 were 93.1, 2.6, 1.8, and 2.5%. Specifically, the PANI-MWCNTs-1 and PANI-MWCNTs-2 show layers of a uniform wrapping/coating of PANI on the tubular surface of the MWCNTs, forming core–shell-like structures.

### 3.2. Electrochemical Characterization of the Sensors Using [Fe(CN)_6_]^3−/4−^

**Figure 4:**
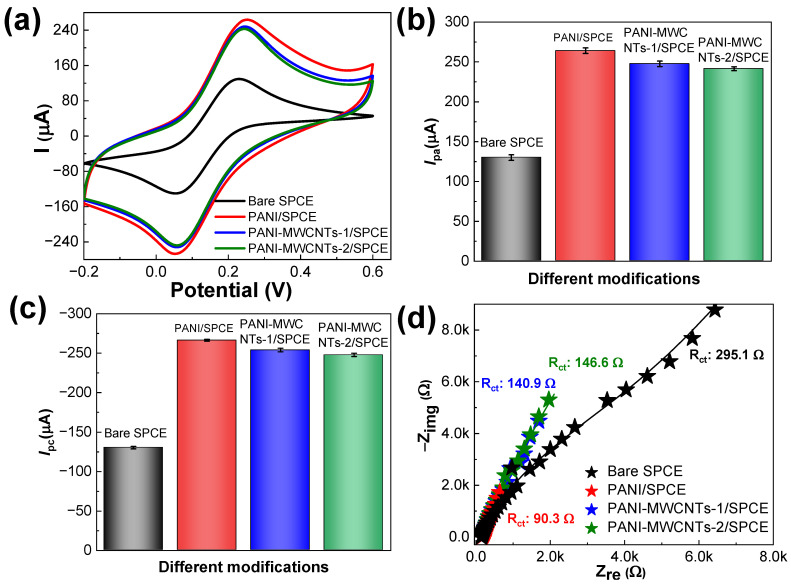
(**a**) Cyclic voltammograms of the sensors in 5.0 mM [Fe(CN)_6_]^3−/4−^ at a scan rate of 50 mV s^−1^, (**b**) the corresponding *I*_pa_ values and (**c**) *I*_pc_ values measured and plotted from (**a**), and (**d**) the electrochemical impedance spectroscopy (EIS) results of the sensors in the same redox probe. Inset is the *R*_ct_ values describing the electron transfer process at each sensor interface. The error bars represent the mean ± s.d. (*n* = 3).

The electrical properties of the bare SPCE, PANI/SPCE, PANI-MWCNTs-1/SPCE, and PANI-MWCNTs-2/SPCE sensors were examined using CV experiments. Figure 4a displays the cyclic voltammetry (CV) results for the sensors in 5.0 mM [Fe(CN)_6_]^3−/4−^ prepared in 0.1 M KCl at 50.0 mV s^−1^. Additionally, the corresponding anodic (*I*_pa_) and cathodic (*I*_pc_) peak currents were measured and are plotted in Figure 4b,c. The *I*_pa_ values for the bare SPCE, PANI/SPCE, PANI-MWCNTs-1/SPCE, and PANI-MWCNTs-2/SPCE were 129.9, 264.2, 247.8, and 241.5 µA, respectively. The *I*_pc_ values for the bare SPCE, PANI/SPCE, PANI-MWCNTs-1/SPCE, and PANI-MWCNTs-2/SPCE were −130.4, −266.4, −254.0, and −247.8 µA, respectively. The bare SPCE reveals well-defined redox peaks. In the CV results, very little current responses were recorded for the bare SPCE, indicating slower electron transfer kinetics. In comparison with the bare SPCE, PANI/SPCE, PANI-MWCNTs-1/SPCE, and PANI-MWCNTs-2/SPCE showed highly increased *I*_pa_ values, illustrating that the PANI and MWCNT modifications improved the electron transport capacity of the bare SPCE sensor. Especially, PANI/SPCE demonstrated the maximum *I*_pa_ value due to the high electroconductive nature of the PANI polymer. In comparison with PANI/SPCE, PANI-MWCNTs-1/SPCE and PANI-MWCNTs-2/SPCE exhibited a slight decrease in *I*_pa_ values due to the loading of MWCNTs into the PANI matrix. However, the *I*_pa_ and *I*_pc_ values of PANI/SPCE were 2.0-fold higher than those of the bare SPCE, while the *I*_pa_ and *I*_pc_ values of PANI-MWCNTs-1/SPCE and PANI-MWCNTs-2/SPCE were 1.9-fold higher than those of the bare SPCE. The Δ*E*_p_ values of PANI/SPCE, PANI-MWCNTs-1/SPCE, and PANI-MWCNTs-2/SPCE were larger than the reversible electron transfer system value (57/n mV). The ratios of *I*_pc_ and *I*_pa_ for the three sensors were closer to −1. These characteristics suggest a quasi-reversible behavior of the sensors in the redox couple, [Fe(CN)_6_]^3−/4−^. The electrochemical active surface area (ECSA) of the sensors was evaluated using the CV technique at different scan rates applying the Randles–Ševčík equation [25]. If the temperature was assumed to be 25 °C, the ECSA values of the bare SPCE, PANI/SPCE, PANI-MWCNTs-1/SPCE, and PANI-MWCNTs-2/SPCE were found to be 0.1250, 0.1376, 0.1358, and 0.1352 cm^2^.

The resistance of the sensors was computed using Nyquist plots obtained at the open-circuit potential with a 5.0 mV alternative current amplitude within the 100 kHz to 0.1 Hz scanning frequency range. Figure 4d depicts that at the sensor interface, diffusion dominates at low frequencies, whereas resistance is found only at higher frequencies. The Nyquist plot of the bare SPCE sensor was best fitted with the equivalent Randles circuit, *R(Q(RW))*, and the plots of the other three modifications were best fitted with *R(C(RW))(CR)*; the fitted parameters are given in Table S1. The formation of the PANI and MWCNT layers necessitates the requirement of additional resistance. The parameters in the equivalent circuits are the solution resistance (R_s_), the charge-transfer resistance (R_ct_), the double-layer capacitance C_CPE_, and the Warburg element capacitance (C_W_) [26]. For the three modifications (PANI/SPCE, PANI-MWCNTs-1/SPCE, and PANI-MWCNTs-2/SPCE), the R_s_ value was slightly changed by <10%, which indicates that the various modifications of the SPCE had no effect at the solution interface. The semi-circular region at higher frequencies corresponds to the process limited by R_ct_, and the linear lower part at lower frequencies corresponds to the diffusion process, indicating the C_W_. The highest *R*_ct_ value around 295.1 Ω corresponds to the bare SPCE. Lower *R*_ct_ values of 90.3, 140.9, and 146.6 Ω were recorded for PANI/SPCE, PANI-MWCNTs-1/SPCE, and PANI-MWCNTs-2/SPCE, respectively. Among these, PANI/SPCE exhibited the lowest resistance. However, PANI-MWCNTs-2/SPCE exhibited reduced *R*_ct_ values compared to the bare SPCE, due to the effective charge-transfer mechanism at the electrode–electrolyte interface. The C_CPE_ values for the bare SPCE, PANI/SPCE, PANI-MWCNTs-1/SPCE, and PANI-MWCNTs-2/SPCE were 1682, 2898, 2764, and 2645 µF cm^−2^, respectively. The magnitudes of changes in the C_W_ and C_CPE_ parameters in the electrolyte solution was strongly influenced by the PANI and MWCNT layers. These results emphasize the fact that the PANI-MWCNTs-2/SPCE composites have remarkable electron transfer properties, which facilitate fast electron-transfer kinetics in comparison with the bare SPCE. Also, the EIS results are in good agreement with the CV results.

The values of charge-transfer resistance, R_ct_ (Ω), obtained from EIS, and ECSA (cm^2^), obtained from the CV analyses, can be utilized to determine the standard heterogeneous rate constant k^0^ (cm/s) of all the sensors using the following equation [27]:

(1) $$k^{0}=\frac{RT}{F^{2}R_{\mathrm{ct}}CA}$$

where k^0^ is the standard heterogeneous electron-transfer rate constant (cm s^−1^), R is the universal gas constant (8.314 J K^−1^ mol^−1^), T is the thermodynamic temperature (298.15 K), F is the Faradays constant (96,485 C mol^−1^), C is the concentration of the [Fe(CN)_6_]^3−/4−^ (0.005 M), and A is the ECSA value for each sensor mentioned in the previous section. Hence, the k^0^ values calculated for the bare SPCE, PANI/SPCE, PANI-MWCNTs-1/SPCE, and PANI-MWCNTs-2/SPCE were 1.4 × 10^−3^, 4.3 × 10^−3^, 2.8 × 10^−3^, and 2.7 × 10^−3^ cm/s, respectively. Higher k^0^ values were recorded for PANI/SPCE, PANI-MWCNTs-1/SPCE, and PANI-MWCNTs-2/SPCE due to the presence of the electroactive PANI. Contrarily, a low k^0^ value was observed for the bare SPCE, indicating a slow electron-transfer rate.

### 3.3. Electrochemical Characterization of the Modified Sensors Using DA

**Figure 5:**
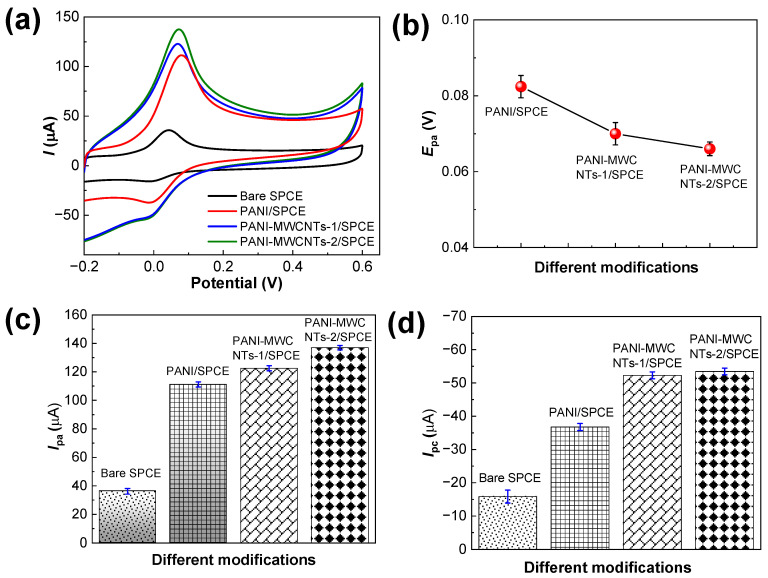
(**a**) CV graphs of the sensors in 0.1 M phosphate-buffered solution (pH 7.0) containing 50.0 µM DA, (**b**) the corresponding *E*_pa_ plot, and the (**c**) *I*_pa_ and (**d**) *I*_pc_ value plots. The error bars represent the mean ± s.d. (*n* = 3).

The oxidation mechanism of DA with the bare SPCE, PANI/SPCE, PANI-MWCNTs-1/SPCE, and PANI-MWCNTs-2/SPCE was studied using the CV experiments. Figure 5a shows the CV results of the sensors towards 50.0 μM DA additions in 0.1 M phosphate-buffered solution with a pH of 7.0. A small redox peak for DA was observed for the bare SPCE, while clear well-distinguished DA redox peaks were recorded for PANI/SPCE, PANI-MWCNTs-1/SPCE, and PANI-MWCNTs-2/SPCE. The slight negative shift of the *E*_pa_ values from +0.082 to +0.066 V evidences the sufficient electrocatalysis of PANI-MWCNTs-2 in Figure 5b.

PANI/SPCE and PANI-MWCNTs-1/SPCE showed increased *I*_pa_ values of 111.2 (a 3.0-fold increase) and 122.6 μA (a 3.3-fold increase) compared to the bare SPCE, whose *I*_pa_ value was 36.2 μA. The *I*_pa_ value generated with PANI-MWCNTs-2/SPCE was 137.0 μA (a 3.8-fold increase) due to the improved catalytic properties of the PANI-MWCNTs-2 nanocomposite (Figure 5c). The *I*_pc_ values of the bare SPCE, PANI/SPCE, PANI-MWCNTs-1/SPCE, and PANI-MWCNTs-2/SPCE were −15.8, −36.8, −52.3, −53.5 μA, respectively. The *I*_pc_ values of PANI-MWCNTs-1/SPCE and PANI-MWCNTs-2/SPCE were closer and approximately 3.3-fold higher than that of the bare SPCE (Figure 5d).

### 3.4. Effect of Scan Rates on PANI-MWCNTs-2/SPCE

**Figure 6:**
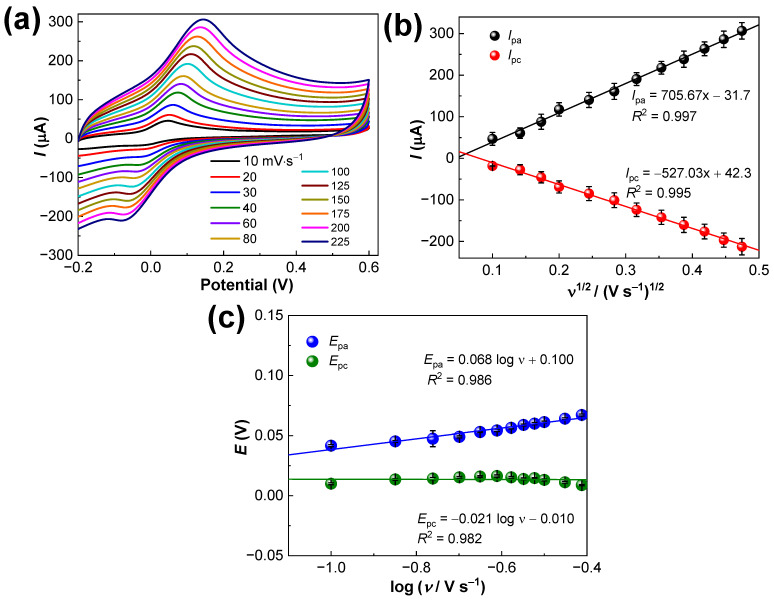
(**a**) CVs of the sensors in 0.1 M phosphate-buffered solution (pH 7.0) containing 10.0 µM DA with increasing scan rates from 10–225 mV·s^−1^, (**b**) *I*_pa_ and *I*_pc_ values plot, and (**c**) the corresponding *E*_pa_ and *E*_pc_ plot. The error bars represent the mean ± s.d. (*n* = 3).

Figure 6a shows the effect of the scan rate (*v*) investigated using CV in 10.0 µM DA. The CV results evince a quasi-reversible electro-oxidation of DA involving the active participation of two electrons (2e^−^). While increasing the scan rates from 10 to 750 mV s^−1^, the *I (I*_pa_ and *I*_pc_) values were found to be increased. The high ion-diffusion rate occurring at higher scan rates resulted in increased *I*_pa_ and *I*_pc_ values, indicating effective chemical interactions between the DA and the PANI-MWCNTs-2/SPCE sensor. As in Figure 6b, the *I*_pa_ values are much higher than the *I*_pc_ values, indicating that the potential sweep favors the electro-oxidation of DA. Figure 6b shows a good linearity between *I* and *v*^½^: *I*_pa_ (µA) = 237.95x − 33.43 (*R*^2^ = 0.986); *I*_pc_ (µA) = 193.59x − 33.38 (*R*^2^ = 0.977), which explains that the redox reaction of DA on the PANI-MWCNTs-2/SPCE sensor is dominantly a diffusion-controlled one.

Moreover, with increases in *v*, the *E*_pa_ and *E*_pc_ values shifted positively. The relationship of *E*_pa_, *E*_pc_, and log *v* are described by Laviron’s equations [28]:

(2) $$E_{pa}=E^{0}+\frac{2.3\;RT}{\left(1-\alpha \right)nF}log\;v$$

(3) $$E_{pc}=E^{0}-\frac{2.3\;RT}{\alpha nF}log\;v$$

(4) $$log\;k_{s}=\alpha log\left(1-\alpha \right)+\left(1-\alpha \right)\;log\;\alpha -log\frac{\;RT}{nFv}-\frac{\left(1-\alpha \right)\alpha nF\Delta E_{p}}{2.3\;RT}$$

where α is the charge-transfer coefficient, *k*_s_ is the standard heterogeneous rate constant, n is the number of electrons involved, and the linear equations of *E*_pa_–log ν and *E*_pc_–log ν were *E*_pa_ = 0.068 log *v* + 0.100 (*R*^2^ = 0.986) and *E*_pc_ = −0.021 log ν—0.01 (*R*^2^ = 0.979), respectively (Figure 6c). The calculated values α = 0.36, *n* = 1.90, and *k*_s_ = 0.24 s^−1^ illustrate that the redox reaction of DA with the PANI-MWCNTs-2/SPCE sensor is entirely diffusion-controlled (*n* = 2) and not a surface-controlled phenomenon. Also, the calculated *k*_s_ value was bigger than those found in previous reports [28,29], suggesting a fast electron transfer of DA on the PANI-MWCNTs-2/SPCE sensor.

### 3.5. Optimization of Solution pH

**Figure 7:**
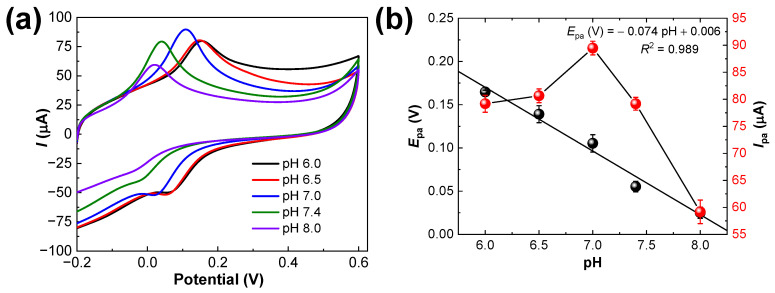
(**a**) CV results for PANI-MWCNTs-2/SPCE in 0.1 M phosphate-buffered solution, pH 7.0, containing 50.0 µM DA, at a scan rate of 50 mV·s^−1^ and (**b**) double plots of *E*_pa_ and *I*_pa_ values measured from the voltammograms in (**a**). The error bars represent the mean ± s.d. (*n* = 3).

The influence of the phosphate-buffered solution’s pH towards 50.0 µM DA on PANI-MWCNTs-2/SPCE was evaluated in the pH range of 6.0−8.0 using CV; the results are shown in Figure 7a. The *E*_pa_ shifted negatively with the increase in pH, indicating the involvement of protons in the electrode reaction. Figure 7b explains the linearity between *E*_pa_ and the pH with the linear regression equation as *E*_pa_ (V) = −0.074 pH + 0.006 (*R*^2^ = 0.989). The slope value was −74.2 mV/pH and the participation of two electrons and two protons (2e^−^; 2H^+^) in the oxidation of DA to a quinone derivative is substantiated. At the same time, when the pH value increased from 6.0 to 7.0, the *I*_pa_ of the DA increased. Nevertheless, when the pH was beyond 7.0, the results showed decreased peak current values. This phenomenon is probably due to the dissociation of the phenolic moiety to produce corresponding anions. Therefore, considering the determination sensitivity (the sharp and distinguished oxidation peak) for DA, a pH 7.0 phosphate-buffered solution was chosen for the subsequent experiments.

### 3.6. Chronoamperometry (CA) Experiments

**Figure 8:**
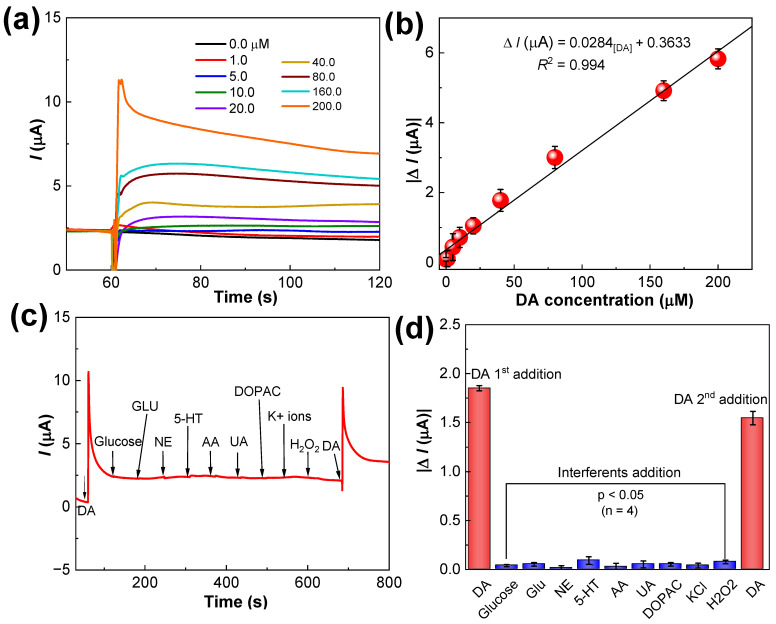
(**a**) CA responses of the PANI-MWCNTs-2/SPCE sensor at the fixed potential of +0.1 V in 0.1 M phosphate-buffered solution, pH 7.0, with additions of different concentrations of DA; (**b**) the linear calibration curve showing the current responses vs. the DA concentration; (**c**) selectivity results of the sensor upon successive additions of 40 μM DA, 12-fold (480.0 μM) higher concentrations of glucose, Glu, NE, 5-HT, AA, and UA; (**d**) the current values measured and plotted with the error bars representing the mean ± s.d. (*n* = 4). *p* values < 0.05 indicate the negligible interferences exerted by the tested interferents.

CA measurements were taken for different concentrations of DA (0.0, 1.0, 5.0, 10.0, 20.0, 40.0, 80.0, 160.0, and 200.0 µM) using PANI-MWCNTs-2/SPCE under optimized conditions. The amperometric responses were stable and proportional to the added DA concentrations (Figure 8a). The responses after 30 s of DA additions were measured and the calibration curve was plotted with the mean values of four measurements (Figure 8b). The linear range was estimated to be 1.0–200.0 µM, and the regression equation was *I* (µA) = 0.0284 C_DA_ (µM) + 0.3633 (R^2^ = 0.994) with a sensitivity of 2.3 µA mM^−1^ cm^−2^. The limit of detection (LOD) was 0.05 µM (S/N = 3). The parameters of our sensor were compared with those of various other electrochemical sensors and are summarized in Table 1. In comparison with the other sensors in Table 1, the LOD of our fabricated sensor is very low. Also, its linear range is wider than that reported in other studies [30,31,32,33,34,35,36,37,38,39,40,41,42,43].

### 3.7. Interference Study

Anti-interference is an important parameter required for the practical application of the PANI-MWCNTs-2/SPCE sensor in real samples. To test the suitability of the proposed sensor in selective DA detection, the influences of other endogenous interferents present in the real samples were studied. The tolerance limit of the sensor is defined as the maximum concentration of the interfering substance that causes an approximately ±5% relative error in the target determination. The results revealed that 12-fold higher concentrations of other endogenous interferents, such as glucose, glutamate (Glu), norepinephrine (NE), serotonin (5-HT), ascorbic acid, and uric acid (480.0 µM), did not show interference in selective DA (40.0 µM) determination. As depicted in Figure 8c, rapid current response was seen only to DA additions, which was not impacted by the tested interferents. The current change generated by these tested interferents are negligible. The first addition of DA at 60 s produced 1.85 µA while its second addition at 700 s produced 1.55 µA responses. The slight decrease in sensitivity is due to the addition of 12-fold excess concentrations of interferents.

Especially, at pH 7.0, glucose and glutamate are near neutral or zero charged and do not donate electron in the electrochemical reactions. Although, NE and 5-HT produce detectable responses, the addition of DA has resulted in a much higher current response than NE and 5-HT, demonstrating the acceptable selectivity of the sensor for DA detection. At pH 7.0, the PANI-MWCNTs-2/SPCE has a net negative charge due to the presence of electronegative sulfide moieties that could repel the negatively charged AA and UA through electrostatic repulsion effects. Briefly, strong electrostatic repulsion between the SO_3_H group in PANI and AA/UA takes place at the sensor interface. In case of positively charged DA, strong electrostatic and hydrogen bonding interactions to electronegative hydroxyl and carboxyl groups of PANI-MWCNTs-2 improve selective DA detection. Hence, the positive-charged DA was more sensitively oxidized on PANI-MWCNTs-2/SPCE whereas negative-charged AA and UA were more inhibited. These results confirm that the unique electrochemical responses of PANI-MWCNTs-2/SPCE to DA molecule are from the special electronic properties of the PANI-MWCNTs-2 material. Similar mechanisms of weakened AA and UA responses in selective DA detection has been reported using a polymer composite of PEDOT and PSS [25].

The CA results shown in Figure 8c were measured and plotted as a bar graph in Figure 8d. The 2nd addition of DA at ~950 s, exhibited similar current responses to 1st addition with only negligible current losses due to other interferents, thus evidencing the superior selectivity of the sensor to DA. The non-interaction of PANI-MWCNTs-2/SPCE with endogenous interferents were also confirmed by the paired *t*-test for the sensors (*n* = 4), which suggest that the PANI-MWCNTs-2/SPCE is highly selective in DA.

### 3.8. Analysis of Ex Vivo Mouse Brain Tissue Homogenates

The functionality of the sensor for selective real-time DA detection was tested in ex vivo mouse brain tissue homogenates from the MPTP induced PD and control models. Since 80% of DA is secreted by the substantia nigra dopaminergic neurons in the brain striatum, the tissue samples were extracted, completely homogenized, and analyzed via the standard addition method.

High levels of DA were recorded in tissue homogenates of the control model, while the PD induced model illustrated ultra-low levels of DA. Since nigrostriatal pathway degeneration is a pathological hallmark of PD, the DA levels were relatively lower than the control model. The results tabulated in Table 2, demonstrated acceptable recovery percentages more than 80%, with a relative standard deviation of less than 4.0%. Hence, the fabricated PANI-MWCNTs-2/SPCE sensor is a one-step rapid and sensitive tool for DA concentration estimation in tissue homogenates.

### 3.9. Reproducibility and Stability

**Figure 9:**
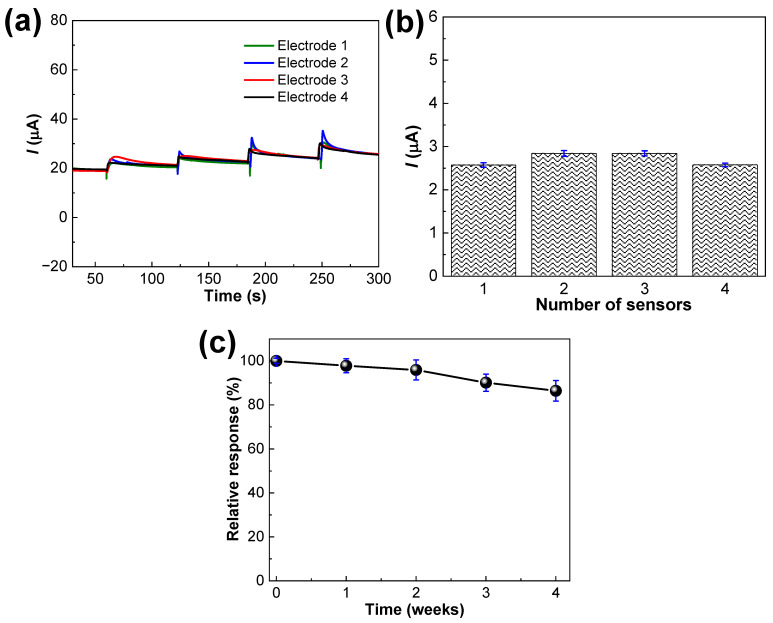
(**a**) Reproducibility of the PANI-MWCNTs-2/SPCE sensor at the fixed potential of +0.1 V in 0.1 M phosphate-buffered solution, pH 7.0 with additions of 50.0 µM concentration of DA, (**b**) the current values plotted from a. (**c**) Stability of the sensors evaluated through 4 weeks of storage at room temperature (25 °C) with the error bars representing the mean ± s.d. (*n* = 3).

Four similarly prepared PANI-MWCNTs-2/SPCE sensors were used to rule out the reproducibility through CA experiments using 50.0 µM DA concentration and the respective amperograms are given in Figure 9a. The resultant current responses of the four similarly prepared sensors are plotted as Figure 9b. Also, the RSD value was 3.2% from four repeated measurements of the four sensors. The stability of the sensors was ruled out after storage at room temperature 25 °C until 4 weeks, evaluated once per week.

The loss of initial current response was 2.2%, 4.1%, 9.9% and 13.6% after 1, 2, 3, and 4 weeks, respectively. After 4 weeks, the sensor had retained nearly 86.4% of the initial response as shown in Figure 9c. Therefore, the sensor exhibited an exceptional reproducibility and stability for DA detection. The effective intermolecular interactions between the PANI and MWCNTs has enhanced the stability of the repeat and long-term measurements.

## 4. Conclusions

Thus, the developed PANI-MWCNTs-2/SPCE sensor was successfully applied to determine DA levels in ex vivo mouse brain homogenates. The sensor demonstrated a sensitivity of 2.3 μA mM^−1^ cm^−2^, a linear range of 1.0–200.0 μM, and an LOD of 0.05 μM in DA detection. The improved electrochemical property of the sensor is attributed to the effective charge-transfer reactions between the MWCNTs and the PANI polymer. Also, the nitrogen and sulfide functionalities in the PANI-MWCNTs-2 provide improved sensitivity and selectivity towards DA, suppressing the impact of susceptible interferents. The sensor remained stable over four weeks of storage and demonstrated high reproducibility. Additionally, the sensor could clearly differentiate DA concentrations in tissue homogenates of both the PD-induced mice and control models. These results suggest that this fabricated PANI-MWCNTs-2/SPCE sensor is a promising diagnostic tool for the study of DA-related diseases like PD. Moreover, the simple fabrication process, increased stability, and selectivity properties pave the way to develop and enhance new diagnostics for target detection in complex biological matrices. The developed sensor can also be used for designing inexpensive wearable biosensors for the detection of other analytes in the future.

## Figures and Tables

**Table 1:** Comparison of parameters obtained from various amperometric DA sensors.

Electrochemical Sensor	Linear Range (µM)	LOD (µM)	Detection Method	Tested Real Samples	Reference
GO/GCE	0.1–15.0	0.27	CA	-	[30]
Graphene/SnO_2_/GCE	1.0–20.0	3.0	DPV	-	[34]
rGO-ZnO/GCE	1.0–70.0	0.33	DPV	Calf plasma and human urine	[32]
Au–SiO_2_/GCE	10.0–100	1.98	DPV	Human serum	[33]
MWCNTs/Q/Nafion/GCE	50.0–500	4.72	CV, LSV	Human serum	[36]
Graphene-Au/GCE	5.0–1000	1.86	CV, DPV	Human serum	[37]
GRNS/PDPA/SiO_2_/GCE	1.0–5.0	0.10	CA	-	[35]
Au–Cu_2_O/rGO/GCE	10.0–90.0	3.9	CV, DPV	Human serum and urine	[38]
GO-PANI/GCE	1.0–14.0	0.50	DPV	-	[39]
Au/PDDA/GNS/GCE	2.0–28.0	1.0	CV, DPV	Human urine	[40]
PG/GCE	0.2–8.0	0.20	CV, DPV	-	[41]
GNP/FTO	30–100	0.22	CV, DPV	Human serum	[42]
PdAu/rGO	1.25–73.75	0.75	CV, DPV	Pharmaceutical samples	[43]
PANI-MWCNTS-2/SPCE	1.0–200	0.05	CV, CA	Ex vivo mouse brain tissue homogenates	This work

GO, graphene oxide; GCE, glassy carbon electrode; SnO_2_, tin (IV) oxide; rGO, reduced graphene oxide; ZnO, zinc oxide; Au, gold; SiO_2_, silica nanoparticles; GRNSs, graphene nanosheets; PDPA, polydiphenylamine; Cu_2_O, copper (I) oxide; PANI, polyaniline; MWCNTs, multi-walled carbon nanotubes; PDDA, poly(dimethyl diallyl ammonium chloride); PG, porous graphene; GNP, graphene nanoplatelet; FTO, fluorine-doped tin oxide electrode; Pd, palladium.

**Table 2:** Comparison of DA concentration (mean of three replicates) in normal and PD induced mice brain tissue homogenates using the CA technique.

Samples	DA Added (µM)	DA Found (µM)	Recovery (%)	RSD (%)
Control model	0.0	0.09	-	1.7
	1.0	0.99	90.8	1.0
	2.0	1.80	86.1	3.9
	3.0	2.60	84.1	2.1
PD induced model	0.0	-	-	0.8
	1.0	0.95	95.0	0.6
	2.0	1.74	87.0	2.1
	3.0	2.43	81.0	0.7

RSD: relative standard deviation.

## Data Availability

Data are contained within the article.

## Supplementary Materials

The following supporting information can be downloaded at https://www.mdpi.com/article/10.3390/bios14060262/s1: Section S1: Instrumentation and measurements; Table S1: EIS parameters obtained by fitting the data to the equivalent electrical circuits of Figure 4d.
