# Supplementary material for: Utilizing a Disposable Sensor with Polyaniline-Doped Multi-Walled Carbon Nanotubes to Enable Dopamine Detection in Ex Vivo Mouse Brain Tissue Homogenates
Source: Biosensors (Basel). 2024 May 21;14(6):262. doi: 10.3390/bios14060262 (PMC11201478; doi:10.3390/bios14060262)
Supplement: Supplementary file 1 [file biosensors-14-00262-s001.zip › biosensors-3018385-supplementary.pdf]

Supplementary material

Utilizing a Disposable Sensor with Polyaniline-Doped Multi-Walled Carbon Nanotubes to Enable Dopamine Detection in Ex Vivo Mouse Brain Tissue Homogenates

Thenmozhi Rajarathinam[1,†], Sivaguru Jayaraman[2†], Jaeheon Seol[3,4], Jaewon Lee[3,4,] and Seung-Cheol Chang[2*]

1 Engineering Research Center for Color-Modulated Extra-Sensory Perception Technology, Pusan National University, Busan 46241, Republic of Korea; thenmozhi@pusan.ac.kr

2 Department of Cogno-Mechatronics Engineering, College of Nanoscience and Nanotechnology, Pusan National University, Busan 46241, Republic of Korea; sivaguru@pusan.ac.kr

3 BIT Convergence-Based Innovative Drug Development Targeting Metainflammation, Department of Pharmacy, College of Pharmacy, Pusan National University, Busan 46241, Republic of Korea; tjfwogjs@pusan.ac.kr (J.S.); neuron@pusan.ac.kr (J.L.)

4 Department of Pharmacy, College of Pharmacy, Pusan National University, Busan 46241, Republic of Korea

*Correspondence: s.c.chang@pusan.ac.kr

†These authors contributed equally to this work.

S1. Instrumentation and Measurements

Field emission scanning electron microscopy (FE-SEM) images were taken using a Zeiss GeminiSEM 500 (Carl Zeiss Microscopy Deutschland GmbH, Oberkochen, Germany). Transmission electron microscopy (FETEM) images were obtained using a Hitachi H-7600 transmission electron microscope (Hitachi High-Tech. Corp., Tokyo, Japan). Cyclic voltammetry (CV) and electrochemical impedance spectroscopy (EIS) measurements were conducted using an electrochemical workstation (model 604E; CH Instruments Inc., Austin, TX, USA). Chronoamperometry (CA) measurements were performed using a potentiostat (Compactstat, Ivium Technologies B.V., Eindhoven, The Netherlands).

A disposable 2.0 mL electrochemical cell was constructed for the CV and EIS measurements. The sensor was initially placed in 2.0 mL of 0.1 M KCl containing a 5.0 mM $[Fe(CN)_6]^{3-/4-}$, and was then scanned from −0.2 to +0.6 V at various scan rates. EIS analysis was performed in the same solution in the frequency range of 100 kHz to 0.1 Hz at a formal potential of 250 mV and an AC amplitude of 5.0 mV. For CA, a 2.0 mL disposable well with phosphate buffer solution was set up, with the sensor being polarized at a fixed potential of +0.1 V. Following the attainment of a stable baseline response, 100 µL of DA solution was introduced and the current responses were measured after 40 s. To construct a calibration curve, measurements were performed several times with various concentrations of DA.

Table S1. EIS parameters obtained by fitting the data to equivalent electrical circuits.

Sensors	R_s (Ω)	R_{CT1} (Ω)	C_W (μF cm^{-2})	C_{CPE} (μF cm^{-2})	R_{CT2} (Ω)	C_{CPE2} (μF cm^{-2})
Bare SPCE	250.5	295.1	1650	1682	-	-
PANI/SPCE	96.28	90.3	1869	2898	63.3	96
PANI-MWCNTs-1/SPCE	102.6	140.9	1676	2764	83.8	190
PANI-MWCNTs-2/SPCE	106.5	146.6	1690	2645	85.2	198
